# Supplementary material for: Maple and hickory leaf litter fungal communities reflect pre-senescent leaf communities
Source: PeerJ. 2022 Jan 27;10:e12701. doi: 10.7717/peerj.12701 (PMC8801177; doi:10.7717/peerj.12701)
Supplement: Supplemental Information 1 — Straight-line distances in meters are between row-column pairs of sites. [file peerj-10-12701-s001.docx]

**Supplemental Table 1. Distances between sample sites.** Straight-line distances in meters are between row-column pairs of sites.

|  | **Site 2** | **Site 3** | **Site 4** | **Site 5** |
| --- | --- | --- | --- | --- |
| **Site 1** | 170.43 | 242.77 | 246.20 | 123.66 |
| **Site 2** | - | 145.00 | 76.29 | 109.95 |
| **Site 3** | - | - | 137.90 | 244.50 |
| **Site 4** | - | - | - | 177.50 |
