# Supplementary material for: Maple and hickory leaf litter fungal communities reflect pre-senescent leaf communities
Source: PeerJ. 2022 Jan 27;10:e12701. doi: 10.7717/peerj.12701 (PMC8801177; doi:10.7717/peerj.12701)
Supplement: Supplemental Information 2 — Included are p-values from permuted pairwise tests. Bolded cell values are comparisons between host species for each substrate. [file peerj-10-12701-s002.docx]

**Table S2. Pairwise PERMANOVA of host species-substrate combinations.** Included are *p*-values from permuted pairwise tests. Bolded cell values are comparisons between host species for each substrate.

| Pairwise PERMANOVA comparisons by host species and substrate | | | | | | | |
| --- | --- | --- | --- | --- | --- | --- | --- |
|  | Acer-Epi | Acer-Lit | Acer-Soil | Carya-Endo | Carya-Epi | Carya-Lit | Carya-Soil |
| Acer-Endo | 0.004 | 0.017 | 0.005 | **0.017** | 0.011 | 0.013 | 0.011 |
| Acer-Epi | - | 0.004 | 0.004 | 0.014 | **0.004** | 0.005 | 0.004 |
| Acer-Lit | - | - | 0.009 | 0.018 | 0.005 | **0.017** | 0.018 |
| Acer-Soil | - | - | - | 0.022 | 0.004 | 0.017 | **0.950** |
| Carya-Endo | - | - | - | - | 0.012 | 0.184 | 0.023 |
| Carya-Epi | - | - | - | - | - | 0.005 | 0.004 |
| Carya-Lit | - | - | - | - | - | - | 0.011 |
