# Supplementary material for: Maple and hickory leaf litter fungal communities reflect pre-senescent leaf communities
Source: PeerJ. 2022 Jan 27;10:e12701. doi: 10.7717/peerj.12701 (PMC8801177; doi:10.7717/peerj.12701)
Supplement: Supplemental Information 3 — Diversity was estimated for Shannon and Inverse Simpson indices using diversity function in vegan. [file peerj-10-12701-s003.docx]

**Supplemental Table 3. ANOVA tables of substrate, host species, and site effects of within-sample diversity.** Diversity was estimated for Shannon and Inverse Simpson indices using diversity function in vegan.

| Shannon Index | | | | | |
| --- | --- | --- | --- | --- | --- |
| Factor | **Df** | **Sum Sq** | **Mean Sq** | **F value** | **Pr(>F)** |
| Substrate | 3 | 273.4 | 91.12 | 8.385 | 0.0002 |
| Host species | 1 | 58.9 | 58.89 | 5.419 | 0.0255 |
| Site | 4 | 102.9 | 25.73 | 2.368 | 0.0704 |
| Residuals | 37 | 402.1 | 10.87 |  |  |
| Inverse Simpson Index | | | | | |
| Factor | **Df** | **Sum Sq** | **Mean Sq** | **F value** | **Pr(>F)** |
| Substrate | 3 | 5.499 | 1.8329 | 4.858 | 0.0060 |
| Host species | 1 | 1.874 | 1.8742 | 4.968 | 0.0320 |
| Site | 4 | 1.56 | 0.39 | 1.034 | 0.4028 |
| Residuals | 37 | 13.959 | 0.3773 |  |  |
