# Supplementary material for: Maple and hickory leaf litter fungal communities reflect pre-senescent leaf communities
Source: PeerJ. 2022 Jan 27;10:e12701. doi: 10.7717/peerj.12701 (PMC8801177; doi:10.7717/peerj.12701)
Supplement: Supplemental Information 4 — Up to 5 indicator taxa with FDR < 0.05 were determined with multipatt for each substrate-host combination. These taxa were identified with the CONSTAX2 classifier against the UNITE database. [file peerj-10-12701-s004.docx]

**Supplemental Table 4. Indicator taxa for combined substrate and host.** Up to 5 indicator taxa with FDR < 0.05 were determined with multipatt for each substrate-host combination. These taxa were identified with the CONSTAX2 classifier against the UNITE database.

| **Substrate** | **Host** | **OTU** | **FDR** | **CONSTAX2 Result** |
| --- | --- | --- | --- | --- |
| **Epiphyte** | *Acer rubrum* | OTU_2 | 0.011 | *Ampelomyces* sp. |
|  |  | OTU_281 | 0.011 | *Ramularia* sp. |
|  |  | OTU_191 | 0.017 | *Taphrina vestergrenii* |
|  |  | OTU_149 | 0.017 | Pseudeurotiaceae sp. |
|  |  | OTU_381 | 0.017 | *Taphrina* sp. |
|  | *Carya ovata* | OTU_56 | 0.011 | *Golubevia pallescens* |
|  |  | OTU_12 | 0.011 | *Erysiphe* sp. |
|  |  | OTU_29 | 0.011 | *Exobasidium* sp. |
|  |  | OTU_68 | 0.011 | *Epicoccum* sp. |
|  |  | OTU_75 | 0.011 | Fungi sp*.* |
| **Endophyte** | *Acer rubrum* | OTU_48 | 0.011 | *Phyllosticta minima* |
|  |  | OTU_83 | 0.011 | *Seimatosporium* sp. |
|  |  | OTU_20 | 0.011 | *Plagiostoma* sp. |
|  |  | OTU_116 | 0.011 | *Angustimassarina acerina* |
|  |  | OTU_461 | 0.017 | *Venturia* sp. |
|  | *Carya ovata* | OTU_332 | 0.011 | *Sphaerulina* sp. |
|  |  | OTU_270 | 0.023 | Ascomycota sp. |
|  |  | OTU_3279 | 0.039 | *Sphaerulina* sp. |
|  |  | OTU_359 | 0.04 | Mycosphaerellaceae sp. |
|  |  | OTU_105 | 0.042 | *Sphaerulina* sp. |
| **Litter** | *Carya ovata* | OTU_50 | 0.011 | Hypocreales sp. |
| **Soil** | *Carya ovata* | OTU_2619 | 0.011 | *Trichoderma* sp. |
|  |  | OTU_2124 | 0.03 | Fungi sp. |
|  |  | OTU_54 | 0.04 | *Mortierella minutissima* |
|  |  | OTU_893 | 0.048 | *Penicillium* sp. |
