# Supplementary figures and images for: Maple and hickory leaf litter fungal communities reflect pre-senescent leaf communities
Source: PeerJ. 2022 Jan 27;10:e12701. doi: 10.7717/peerj.12701 (PMC8801177; doi:10.7717/peerj.12701)

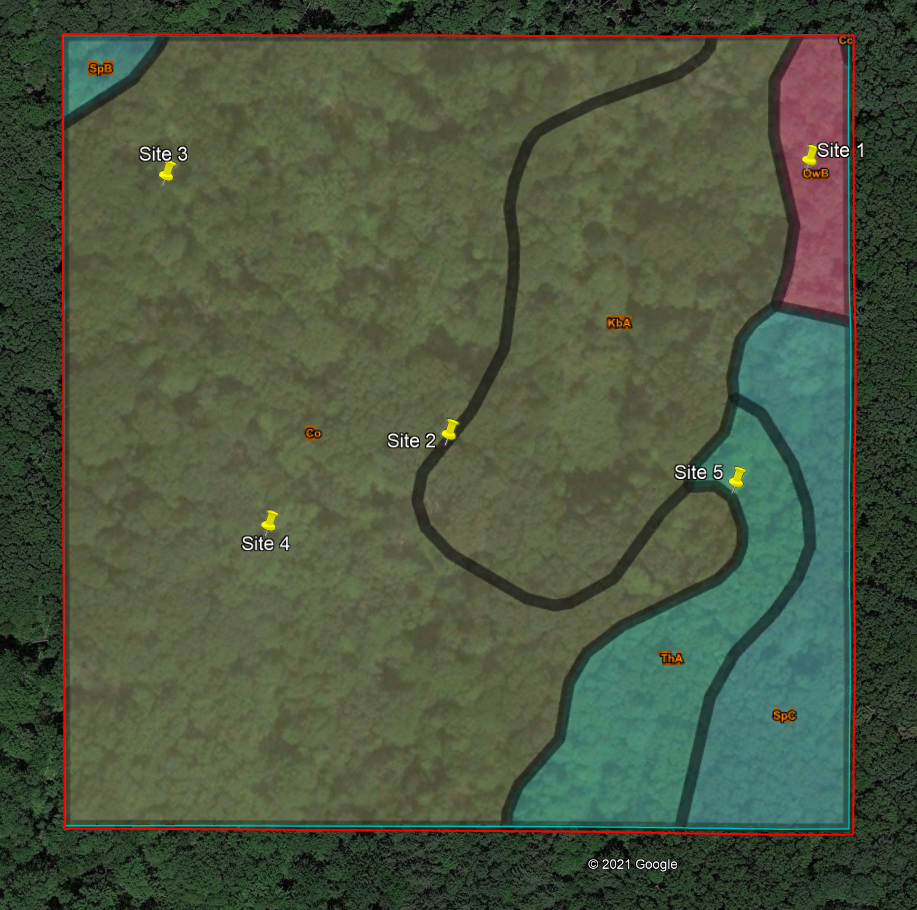

Supplement: Supplemental Information 5 — (Co) Colwood-Brookston loams; (KbA) Kibbie loam, 0 to 3 percent slopes; (OwB) Owosso-Marlette sandy loams, 2 to 6 percent slopes; (SpB) Spinks loamy sand, 0 to 6 percent slopes; (SpC) Spinks loamy sand, 6 to 12 percent slopes, (ThA) Thetford loamy sand, 0 to 3 percent slopes. Map data © 2021 Google” [file peerj-10-12701-s005.png]

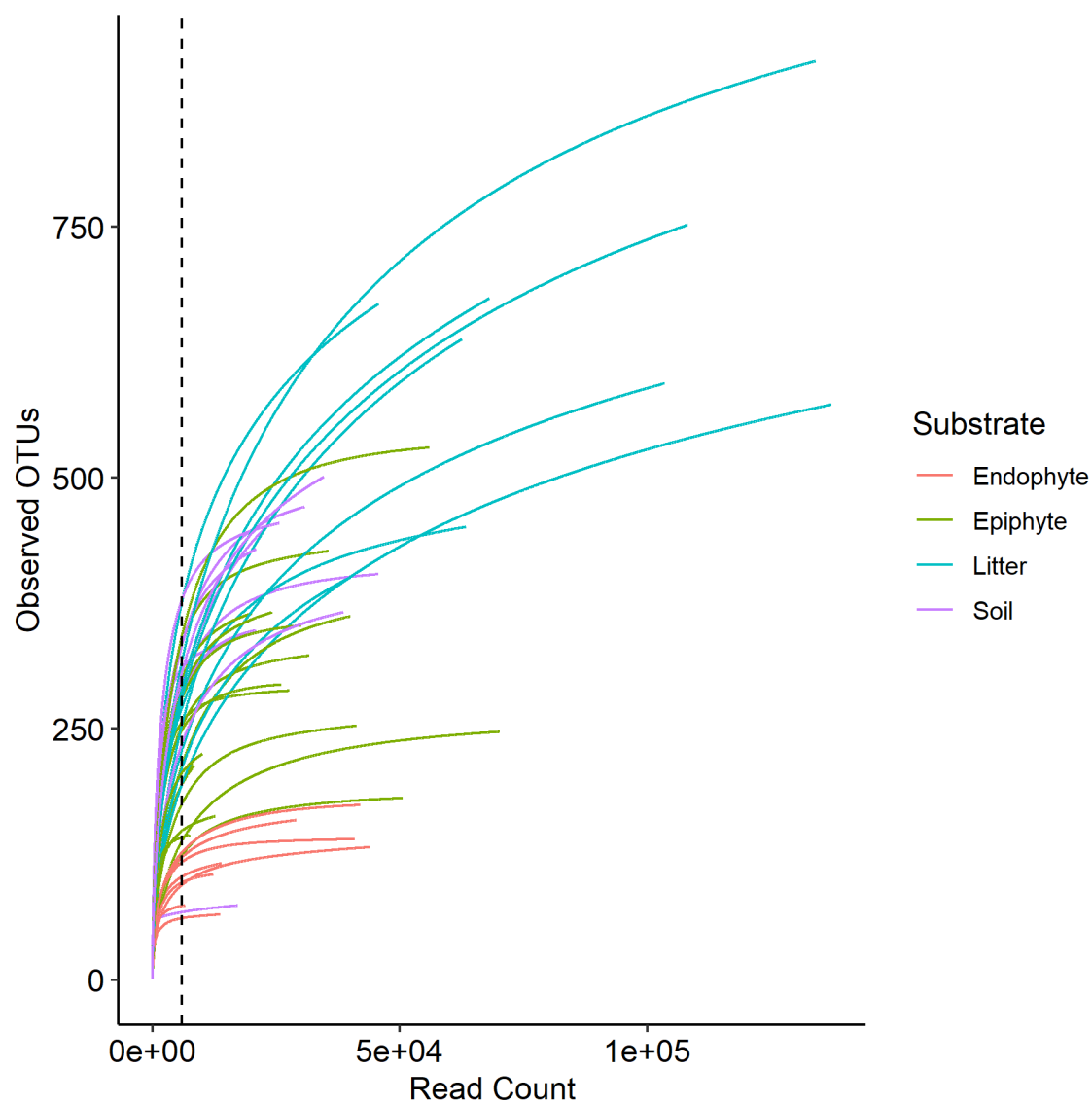

Supplement: Supplemental Information 6 — Curves were determined using “rrarefy” in vegan to assess rarefaction depth to use for downstream analyses. A vertical line is positioned at the applied rarefaction depth. [file peerj-10-12701-s006.pdf]

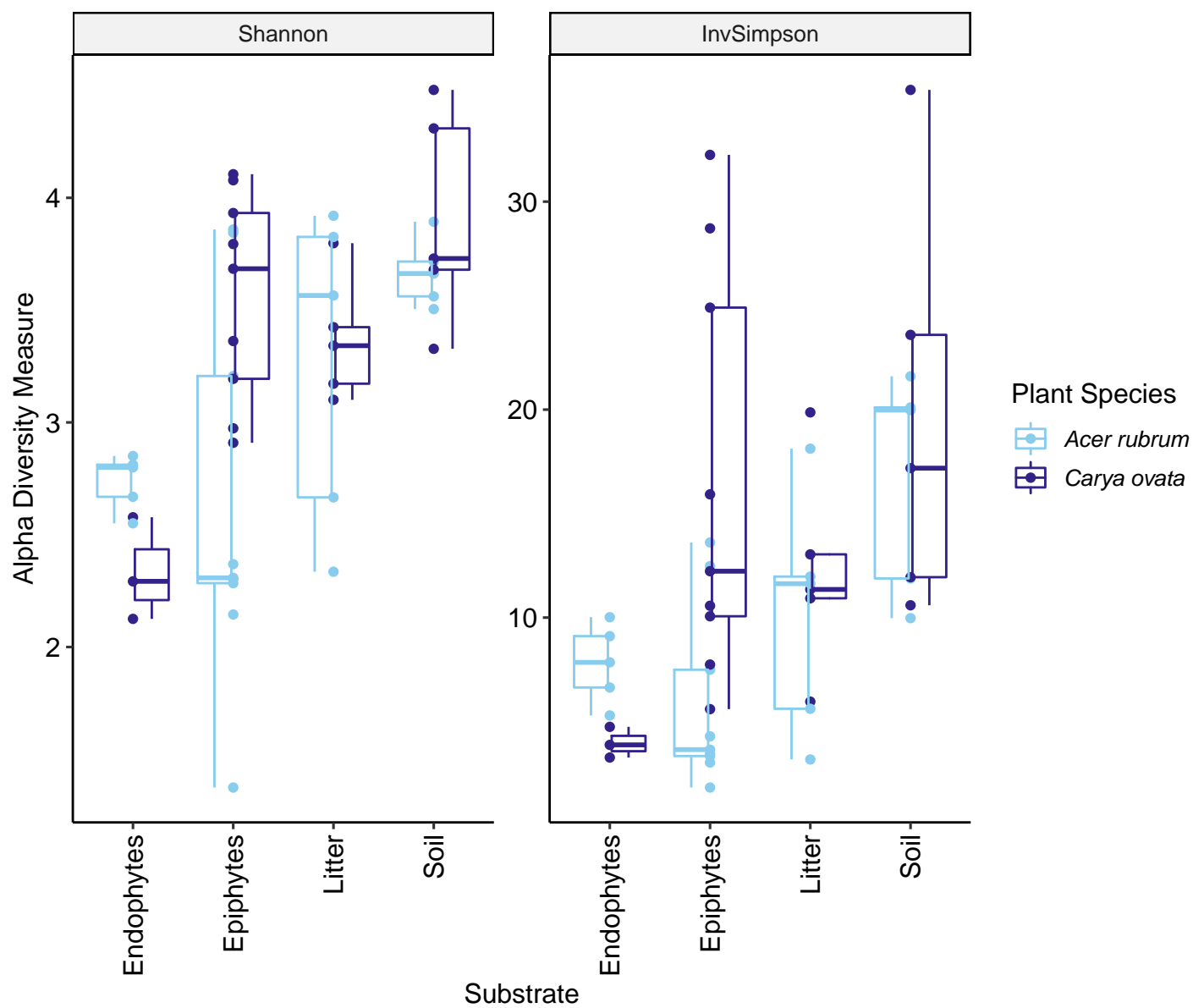

Supplement: Supplemental Information 7 — Shannon and Inverse Simpson estimates of within-sample (alpha) diversity for all samples, grouped by substrate and host species. Diversity estimates were determined using the phyloseq package. [file peerj-10-12701-s007.pdf]

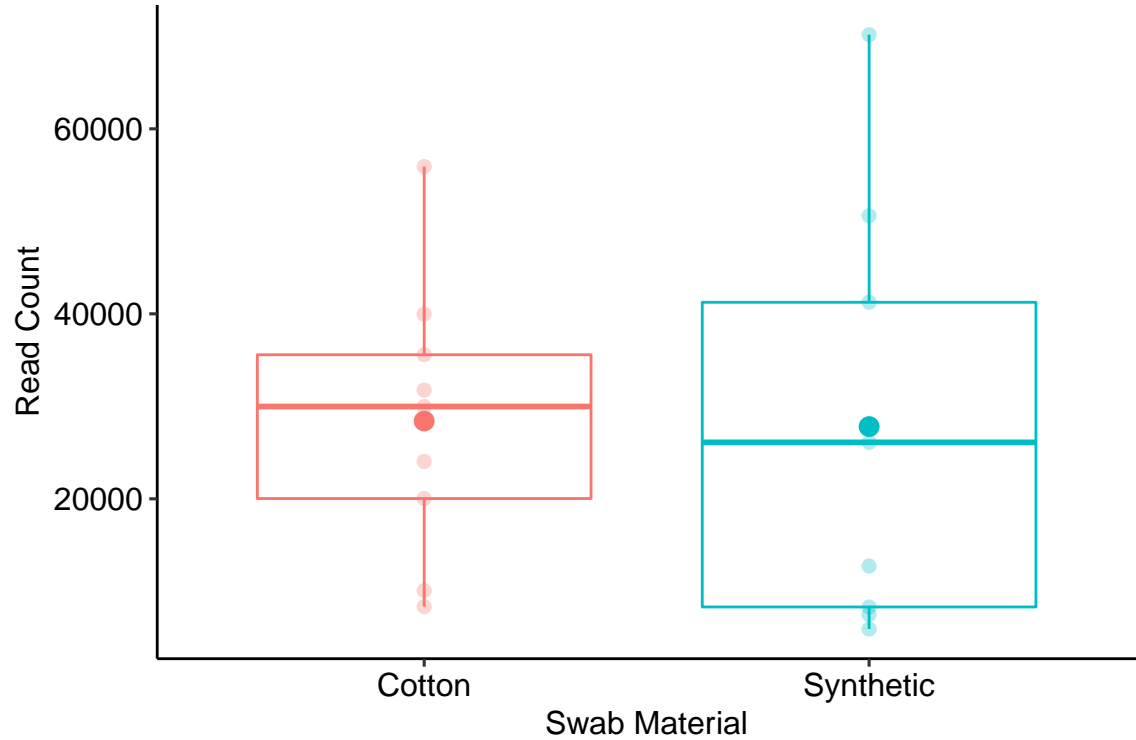

Supplement: Supplemental Information 9 — Read counts prior to rarefaction were compared between swab material types. Means are displayed with a large solid circle. [file peerj-10-12701-s009.pdf]
