# Supplementary material for: Maple and hickory leaf litter fungal communities reflect pre-senescent leaf communities
Source: PeerJ. 2022 Jan 27;10:e12701. doi: 10.7717/peerj.12701 (PMC8801177; doi:10.7717/peerj.12701)
Supplement: Supplemental Information 8 — Heatmap coloration shows the Pearson r coefficient between samples using rarefied OTU counts. [file peerj-10-12701-s008.pdf]

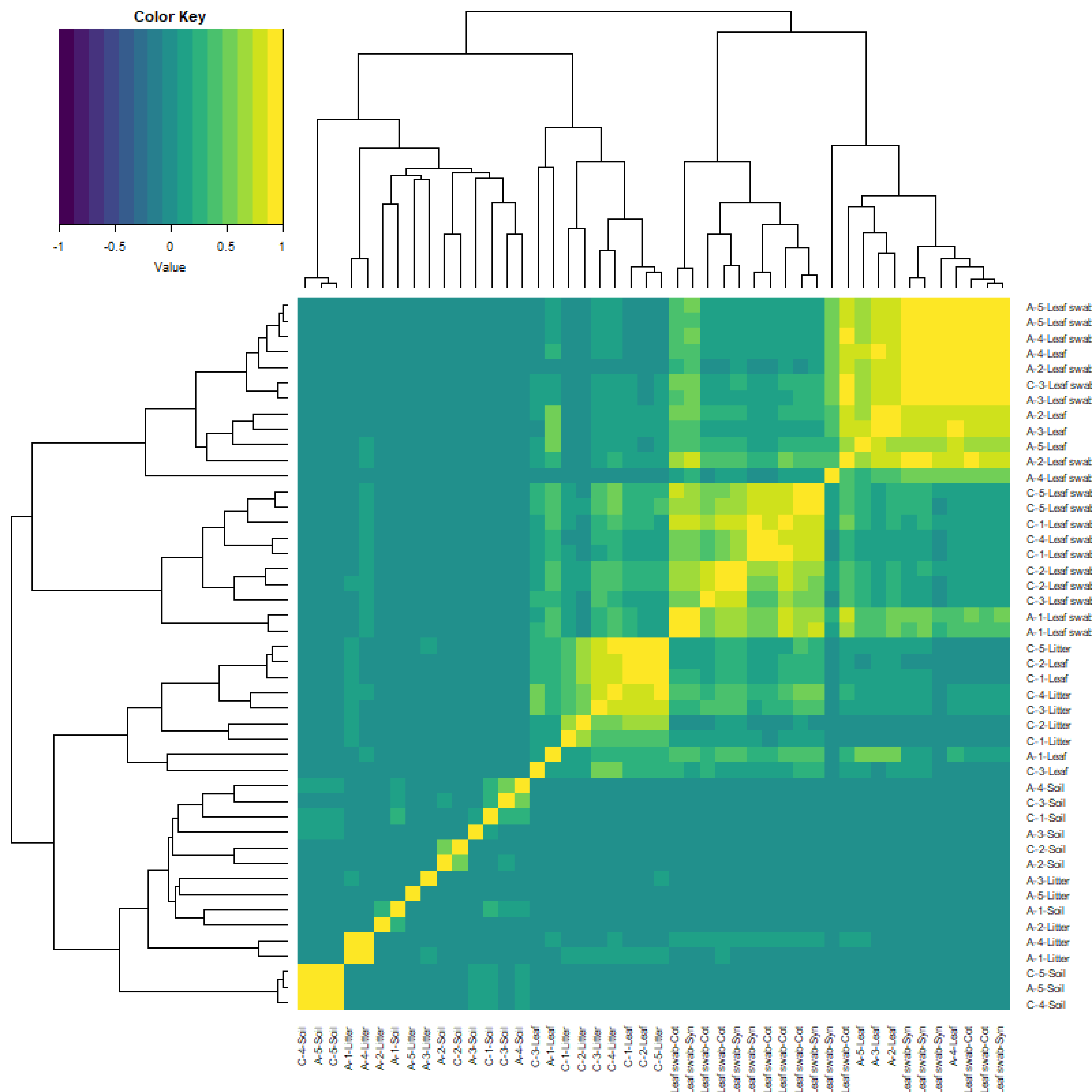

Color Key
-1
-0.5
0
0.5
1
Value
A-5-Leaf swab
A-5-Leaf swab
A-4-Leaf swab
A-4-Leaf
A-2-Leaf swab
C-3-Leaf swab
A-3-Leaf swab
A-2-Leaf
A-3-Leaf
A-5-Leaf
A-2-Leaf swab
A-4-Leaf swab
C-5-Leaf swab
C-5-Leaf swab
C-1-Leaf swab
C-4-Leaf swab
C-1-Leaf swab
C-2-Leaf swab
C-2-Leaf swab
C-3-Leaf swab
A-1-Leaf swab
A-1-Leaf swab
C-5-Litter
C-2-Leaf
C-1-Leaf
C-4-Litter
C-3-Litter
C-2-Litter
C-1-Litter
A-1-Leaf
C-3-Leaf
A-4-Soil
C-3-Soil
C-1-Soil
A-3-Soil
C-2-Soil
A-2-Soil
A-3-Litter
A-5-Litter
A-1-Soil
A-2-Litter
A-4-Litter
A-1-Litter
C-5-Soil
A-5-Soil
C-4-Soil
C-4-Soil
A-5-Soil
C-5-Soil
A-1-Litter
A-4-Litter
A-2-Litter
A-1-Soil
A-5-Litter
A-3-Litter
A-2-Soil
C-2-Soil
A-3-Soil
C-1-Soil
C-3-Soil
A-4-Soil
C-3-Leaf
A-1-Leaf
C-1-Litter
C-2-Litter
C-3-Litter
C-4-Litter
C-1-Leaf
C-2-Leaf
C-5-Litter
A-5-Leaf
A-3-Leaf
A-2-Leaf
A-4-Leaf
